# *Madurella mycetomatis* Is Highly Susceptible to Ravuconazole

**DOI:** 10.1371/journal.pntd.0002942

**Published:** 2014-06-19

**Authors:** Sarah Abdalla Ahmed, Wendy Kloezen, Frederick Duncanson, Ed E. Zijlstra, G. Sybren de Hoog, Ahmed H. Fahal, Wendy W. J. van de Sande

**Affiliations:** 1 Faculty of Medical Laboratory Sciences, University of Khartoum, Khartoum, Sudan; 2 Centraalbureau voor Schimmelcultures CBS-KNAW Fungal Biodiversity Centre, Utrecht, The Netherlands; 3 Institute for Biodiversity and Ecosystem Dynamics, University of Amsterdam, The Netherlands; 4 Erasmus MC, Department of Medical Microbiology and Infectious Diseases, Rotterdam, The Netherlands; 5 Eisai Inc., Woodcliff Lake, New Jersey, United States of America; 6 Rotterdam Centre for Tropical Medicine, Rotterdam, The Netherlands; 7 Peking University Health Science Center, Research Center for Medical Mycology, Beijing, China; 8 Sun Yat-Sen Memorial Hospital, Sun Yat-Sen University, Guangzhou, China; 9 Shanghai Institute of Medical Mycology, Changzheng Hospital, Second Military Medical University, Shanghai, China; 10 Basic Pathology Department, Federal University of Paraná State, Curitiba, Paraná, Brazil; 11 King Abdulassiz University, Jeddah, Saudi Arabia; 12 Mycetoma Research Centre, University of Khartoum, Khartoum, Sudan; Fundação Oswaldo Cruz, Brazil

## Abstract

The current treatment of eumycetoma utilizing ketoconazole is unsatisfactory because of high recurrence rates, which often leads to complications and unnecessary amputations, and its comparatively high cost in endemic areas. Hence, an effective and affordable drug is required to improve therapeutic outcome. E1224 is a potent orally available, broad-spectrum triazole currently being developed for the treatment of Chagas disease. E1224 is a prodrug that is rapidly converted to ravuconazole. Plasma levels of E1224 are low and transient, and its therapeutically active moiety, ravuconazole is therapeutically active. In the present study, the *in vitro* activity of ravuconazole against *Madurella mycetomatis,* the most common etiologic agent of eumycetoma, was evaluated and compared to that of ketoconazole and itraconazole. Ravuconazole showed excellent activity with MICs ranging between ≤0.002 and 0.031 µg/ml, which were significantly lower than the MICs reported for ketoconazole and itraconazole. On the basis of our findings, E1224 with its resultant active moiety, ravuconazole, could be an effective and affordable therapeutic option for the treatment of eumycetoma.

## Author Summary

*Madurella mycetomatis* is the most common etiologic agent of eumycetoma worldwide. Treatment of this infection is very difficult and associated with high recurrence rates and low cure rates. Currently the treatment consists of a combination of surgery and antifungal therapy. Antifungal therapy is usually given for at least one year. However, the commonly used antifungal ketoconazole is too expensive for many patients in endemic countries and has many side effects. In the present study we evaluated the *in vitro* activity of the new antifungal agent ravuconazole against *M. mycetomatis*. The drug showed excellent *in vitro* activity against all tested strains and its prodrug, E1224, might be a potential new therapeutic option for eumycetoma caused by *M. mycetomatis.*

## Introduction

Mycetoma is a serious health problem with high morbidity. It is endemic in subtropical areas and often leads to severe deformity and disability [1]. The disease has long been disregarded by international health organizations but was recently recognized by WHO as a neglected tropical condition (http://www.who.int/neglected_diseases/diseases/en/). One of the main problems of eumycetoma is its recalcitrant nature, which necessitates prolonged antifungal therapy combined with massive and repeated surgical debridement. In severe cases, amputation of the affected part may be the only remaining treatment option [2]. *Madurella mycetomatis* is the most common fungal pathogen causing eumycetoma in arid climate zones, particularly in northeastern Africa. The infection by *M. mycetomatis* is characterized by the presence of black grains in tissue [3]. Previous reports showed that this fungus was most susceptible to the azole class of antifungal agents [4], [5], [6]. Ketoconazole and itraconazole are the most frequently used drugs for the treatment of mycetoma. However, therapy failure is common and high recurrence and amputation rates are reported [7]. Another concern is that both the Food and Drug Administration (FDA) and the European Medicines Agency (EMEA) recently restricted the use of ketoconazole due to its toxic side effects (http://www.fda.gov/Drugs/DrugSafety/ucm362415.htm) [8], making the need for an alternative treatment for eumycetoma even more urgent.

Since *M. mycetomatis* appeared to be most susceptible to the azole class of antifungal agents, a new azole probably has the best chance of meeting that need. A new azole currently under development is ravuconazole. Ravuconazole is a broad-spectrum triazole that showed activity against a wide array of fungal species including *Aspergillus* spp., *Candida* spp., and *Cryptococcus neoformans* [9], [10]. Studies have shown that the efficacy of this new triazole was comparable to that of posaconazole and voriconazole [9], [10], [11]. In addition to antifungal activity, ravuconazole also showed *in vitro* activity against the parasite *Trypanosoma cruzi,* the causative agent of Chagas disease, another neglected tropical disease on the WHO list [12]. Eisai developed a prodrug of ravuconazole (E1224) which has a simpler chemical structure, is safe, and has a long half-life in humans [13]. These attributes will reduce the costs of ravuconazole treatment and will make it an affordable drug for people in endemic countries. In the present study, we investigated the antifungal activity of ravuconazole (the active moiety of E1224) against 23 isolates of *Madurella mycetomatis*.

## Materials and Methods

### Fungal isolates

The 23 isolates were obtained from 23 patients seen at the Mycetoma Research Centre, University of Khartoum, Sudan, and preserved in the collection of Erasmus Medical Centre, Rotterdam, and CBS (Fungal Biodiversity Centre), Utrecht, The Netherlands. All the strains were previously collected and were taken from the above mentioned collections for the study. The identity of the strains was confirmed with a multi-locus analysis of rDNA internal transcribed spacer and partial large subunit and compared with *M. mycetomatis* type strain CBS 109801 [14]. Prior to susceptibility testing, fresh cultures of the strains were made on Sabouraud's dextrose agar (SDA) plates which were incubated for three weeks at 37 °C.

### *In vitro* susceptibility testing

The *in vitro* activity of ravuconazole was determined using the 2,3-bis (2-methoxy-4-nitro-5-sulfophenyl)-5-[(phenylamino) carbonyl]-2H-tetrazolium hydroxide (XTT) broth micro-dilution assay to estimate the minimum inhibitory concentration (MIC) for the strains [15]. The method was described and validated by Ahmed et al. for susceptibility testing of *M. mycetomatis* using a standardized hyphal inoculum [15]. For the assay, about 2 cm of fungal colonies grown on SDA plates were scraped off and inoculated into tubes with 10 ml RPMI 1640 medium containing 0.35 g/liter L-glutamine and 1.98 mM 4-morpholinepropanesulfonic acid (MOPS). Prior to incubation, the fungal mass was sonicated for 5 s at the maximum power of a sonicator (Beun de Ronde, The Netherlands). Tubes were incubated for 7 days at 37 °C. After incubation, mycelia were washed once with RPMI and sonicated again for 5 s at the maximum power. The final inocula were adjusted spectrophotometrically (660 nm; Novaspec II, Pharmacia Biotech, Cambridge, U.K.) to obtain transmissions in the range of 69–71%. Ravuconazole was kindly provided by Eisai Co., Ltd., as reagent-grade powder and used in concentrations ranging from 0.002 to 2 µg/ml. In addition to ravuconazole, MICs were also determined for ketoconazole (Janssen Pharmaceuticals, Belgium) and itraconazole (Janssen) in concentrations ranging from 0.016 to 16 µg/ml. The assay was carried out in round-bottom microtitre plate where 100 µl of the inoculum were added to 2 µl of drug concentrations. For each isolate drug free control and negative control were included to define the end point reading. Endpoint reading was done after 7 days of incubation at 37 °C using XTT; MICs were defined as the lowest concentration with a minimum of 80% growth reduction. With the XTT assay, 100% reduction in viable fungal mass could not be used as an end-point, since a number of strains had pigments that influenced the color intensity [15]. The 80% boundary was found to correspond with the MICs obtained visually for the fungistatic drug amphotericin B [15]. All experiments were performed in duplicate on different days. Association between MICs obtained for ravuconazole and the comparator azoles were done using the Mann-Whitney test and Wilcoxon's signed rank test.

## Results

**Figure 1:**
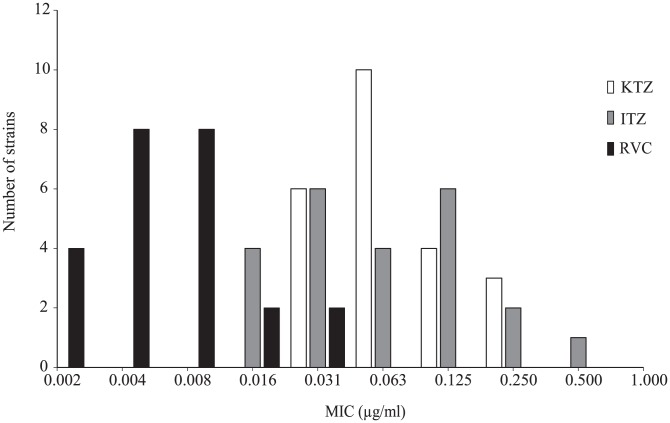
*In vitro* activities of ketoconazole (KTC), itraconazole (ITC), and ravuconazole (RVC) against 23 isolates of *Madurella mycetomatis* represented by MICs.

As shown in Table 1, fifty percent of the strains were inhibited by a concentration of 0.063 µg/ml (MIC_50_) for both ketoconazole and itraconazole, while a concentration of 0.25 µg/ml (MIC_90_) was required to inhibit 90% of the strains. Significantly lower MICs were obtained with ravuconazole in comparison to ketoconazole and itraconazole (Mann-Whitney, p<0.0001 for both comparisons), with MICs ranging from ≤0.002 to 0.031 µg/ml (Fig. 1). Same results were obtained when using Wilcoxon's signed rank test [Z-value: -4.1973, p-value is 0.00 for both drugs]. Moreover, there is no cross susceptibility among strains showed low MICs for ravuconazole and those of ketoconazole and itraconazole. A concentration of 0.004 µg/ml ravuconazole was needed to inhibit 50% of the strains, whereas 0.016 µg/ml was required to inhibit 90% of them.

**Table 1:** *In vitro* susceptibility of *Madurella mycetomatis* to ketoconazole, itraconazole, and ravuconazole.

Antifungal agent	GM^a^ MIC (μg/ml)	MIC Range (μg/ml)	MIC_50_ (μg/ml)	MIC_90_ (μg/ml)
Ketoconazole	0.072	0.031–0.25	0.063	0.25
Itraconazole	0.063	≤0.016–0.5	0.063	0.25
Ravuconazole	0.005	≤0.002–0.031	0.004	0.016

a GM, geometric mean.

## Discussion

In this study, we demonstrated that *Madurella mycetomatis,* the most common etiologic pathogen for mycetoma, was highly susceptible to ravuconazole with MICs ranging from ≤0.002 to 0.031 µg/ml. These MICs were not only considerably lower than those found for ketoconazole and itraconazole in the present study, but they were also lower than those reported for voriconazole (0.016–1 µg/ml), posaconazole (0.03–0.125 µg/ml), and isavuconazole (0.016–0.125 µg/ml) [4], [5], [6]. Only a few reports are available regarding the susceptibility of other eumycetoma causative agents towards ravuconazole. Studies have shown that ravuconazole has inhibitory activity against the black-grain eumycetoma species *Exophiala jeanselmei* and to the saprobe *Curvularia lunata* that occasionally has been observed in eumycetoma [10], [16]. In contrast, resistance was reported for the white-grain eumycetoma causative pathogens *Pseudallescheria boydii* and *Fusarium* species [10], [16], [17]. Good inhibitory activity of ravuconazole was reported for members of *Chaetomium*, a genus that was found to be phylogenetically close to the genus *Madurella* [14], [18]. Low MICs were reported for *Chaetomium* species ranging from 0.06 to 1 µg/ml, but these values were higher than the results reported in this communication [18]. Studies of the *in vitro* activity of ravuconazole against the more common pathogenic fungi, including *Cryptococcus neoformans*, *Candida* species, *Aspergillus* species, and the dermatophytes, showed that the drug has activity comparable to that of other triazoles [10], [16], [19], [20]. Moreover, ravuconazole showed potent *in vitro* activity against the parasite *Trypanosoma cruzi* [12]. Several studies have been conducted to evaluate the *in vivo* efficacy of ravuconazole and E1224 using animal models of aspergillosis, candidiasis, and cryptococcosis, with each demonstrating encouraging activity of the drug [21], [22], [23]. In addition, phase 1/2 clinical trials have shown that ravuconazole and E1224 were well tolerated. Ravuconazole had a relatively long half-life of 4–8 days and the peak plasma concentrations of the drug ranged from 1.20 to 6.02 µg/ml when 50–400 mg/day was administrated orally for 14 days [24]. E1224 provides the advantage of more favorable pharmacokinetics with a half-life of ravuconazole (resulting from conversion of E1224 to ravuconazole) of 7.7 to 10.5 days and peak plasma levels of 3.7–379 µg/ml when 200–400 mg/day was administrated orally for 14 days [25]. This serum level of the drug is much higher than the concentration needed to inhibit 90% of the *M. mycetomatis* strains in the present study (MIC_90_ of 0.016 µg/ml). Furthermore, in rabbits it was demonstrated that ravuconazole concentrations in the liver, adipose tissue, marrow, kidney, lung, brain and spleen exceeded concurrent plasma concentrations [26]. Moreover, high concentrations were also detected in lung and uterus of rat [27]. Due to these high levels of the drug in tissue, good therapeutic efficacy was obtained in animal models with pulmonary and disseminated aspergillosis, candidiasis, histoplasmosis, intracranial and disseminated cryptococcosis [21], [23], [28], [29]. Based on the *in vitro* susceptibility generated in this study, the next step will be to study the efficacy of ravuconazole in an animal model of mycetoma.

We conclude that ravuconazole has potent *in vitro* activity against *M. mycetomatis*. Compared to other infectious fungi, *Madurella* is exceptionally susceptible to this drug. With its favorable pharmacokinetic properties and low toxicity, E1224 with its resultant active moiety, ravuconazole, could be a promising antifungal agent for treatment of eumycetoma. A clinical trial is now required for an *in vitro*-*in vivo* correlation of the activity of the drug.
